# Book Notices

**Published:** 1878-04-20

**Authors:** 


### BOOK NOTICES.

CONTRIBUTIONS to the History of Medical Education and Medical Institutions in the United States, 1776–1877.

Special report prepared for the United States Bureau of Education, by N. S. Davis, A. M. M. D., Washington, D. C.

ORIGIN and Progress of Medical Jurisprudence—1876 to 1777.

A centennial address, by Stanford E. Chaille, A. M., M.D. From Transactions of the International Medical Congress for the benefit of the legal and medical professions of the United States.

SUSPENSION as a Means of Treating Spinal Distortions. By Benjamin Lee, A. M. M. D.: Philadelphia. From Transactions American Medical ssociation.

ETIOLOGY of Intemperance. By Charles W. Earle, M. D., Physician to the Washington Home, Chicago.

SCARLATINA IN CHICAGO, particularly the Epidemic of 1876–7. By Chas. W. Earle, M. D., Chicago.

"THE BLOOD IS THE LIFE." A Treatise on Immortality, founded on Bible truths. By Joseph Wheeler, of Bath, New York.

METEOROLOGY in the Science of Medicine: An address delivered before the Austrian Meteorological Society, by Dr. Schreiber. Translated from the *Oesterreichische Zeitschrift fur Meteorologie* by W. H. Geddings, M. D., of South Carolina.

ANNUAL REPORT of the Pennsylvania Free Dispensary for Skin Diseases. Philadelphia.

ATLAS OF SKIN DISEASES, by Louis A. Duhring, M.D., Professor of Skin Diseases in the Hospital of the University of Pennsylvania. Physician to the Dispensary for Skin Diseases, Philadelphia, etc.

Part third, containing beautiful and life-like illustrations of—

Eczema—(Squamosum).

Syphiloderma—(Erythematosum).

Purpura—(Simplex).

Syphiloderma—(Papulosum).

These illustrations are painted from life. The work will appear in parts to be issued quarterly, each containing four plates, royal quarto in size. The work will be completed in eight or ten parts, and will constitute the most valuable aid to the practitioner in the diagnosis of skin diseases that has, perhaps, ever yet been offered the professon.

Price, $2 50 per part. J. Lippincott & Co., Philadelphia.

---

THYROID GLAND REMOVED.—Dr. O. E. Newton, of Cincinnati, recently performed this rare and bloody operation successfully.

---

DIED.—One of our subscribers, Dr. J. E. Borroughs, of Stevensville, Texas, died on October 2d, 1877.

---

THE Pharmaceutical Association of Georgia met in Augusta, on the 9th inst. Dr. P. H. Sand, the President, delivered an address. Resolutions of regret at the death of Dr. J. A. Taylor were adopted and an eloquent eulogy delivered by Mr. Rankin, of Atlanta.

Laws of pharmacy were discussed and agreed upon—to be submitted to next Legislature. Mr. J. Zacharius will be the orator, and Savannah the place of next meeting.

